# Interactions between Bile Acids and Nuclear Receptors and Their Effects on Lipid Metabolism and Liver Diseases

**DOI:** 10.1155/2012/560715

**Published:** 2012-04-22

**Authors:** David Q.-H. Wang, Brent A. Neuschwander-Tetri, Piero Portincasa, William M. Pandak

**Affiliations:** ^1^Division of Gastroenterology and Hepatology, Department of Internal Medicine, Edward Doisy Research Center, Saint Louis University School of Medicine, 1100 S. Grand Boulevard, St. Louis, MO 63104, USA; ^2^Division of Gastroenterology and Hepatology, Saint Louis University School of Medicine, 3635 Vista Avenue, St. Louis, MO 63110, USA; ^3^Clinica Medica “A. Murri”, Department of Interdisciplinary Medicine (DIM), University of Bari School of Medicine, Policlinico Hospital, Piazza G. Cesare 11, 70124 Bari, Italy; ^4^Division of Gastroenterology and Hepatology, Department of Medicine, Virginia Commonwealth University Health Sciences Center & Veterans Affairs Medical Center, P.O. Box 980341, Richmond, VA 23298, USA

In this special issue of *Journal of Lipids*, we acknowledge the contributions by several experts offering timely perspectives on the complex interactions between bile acids and nuclear receptors (NRs) on lipid metabolism and liver diseases at different levels and contexts in the body.

NRs are found within the interior of cells and are defined as ligand-activated transcriptional regulators of several key aspects of body physiology and pathophysiology. NRs regulate gene transcription through interaction with cellular coactivators and corepressors. In the liver, NRs play a key role in a large variety of metabolic processes such as cholesterol, bile acid, fatty acid, and glucose homeostasis, as well as drug disposition. Also, additional critical processes involving the pathophysiology of liver diseases—inflammation and fibrosis, regeneration, cell differentiation, and tumor formation—are modulated by NRs. Of note, NRs are or might soon become drug targets. Despite the huge accumulation of knowledge in the field, the true comprehension of interactions between bile acids and NRs on lipid metabolism and hepatobiliary diseases has remained elusive. Thus continuous efforts are being made to understand the molecular functions of NRs, the significance of bile acid-controlled signaling pathways, and interactions of NRs on a number of metabolic and hepatic diseases. 

The paper of T. Li and Y. L. Chiang is focused on the role of bile acid signaling in the regulation of glucose and lipid metabolism. Besides their detergent properties and key physiological functions, bile acids are also acting as potent metabolic regulators of glucose and lipid homeostasis. The identification of bile acid-activated nuclear receptor farnesoid X receptor (FXR) and cell surface G-protein-coupled receptor TGR5 has significantly advanced our understanding on how bile acid signaling regulates cellular metabolism in health and disease. Thus, novel therapeutic strategies can be envisioned which target bile acid metabolism for the treatment of metabolic disorders such as obesity, insulin resistance, and the metabolic syndrome.

NRs comprise one of the most abundant classes of transcriptional regulators of metabolic diseases and have emerged as promising pharmaceutical targets. The paper by G. Garruti et al. deals with the myriad roles of small heterodimer partner (SHP), a unique orphan nuclear receptor lacking a DNA-binding domain, but containing a putative ligand-binding domain. About half of mammalian NRs and several transcriptional coregulators can interact with SHP. SHP is a transcriptional regulator affecting multiple key biological functions and metabolic processes including cholesterol, bile acid, and fatty acid metabolism, as well as reproductive biology and glucose-energy homeostasis. In humans, studies are emerging on the association of SHP genetic variation with birth weight, high body mass index, obesity, insulin resistance, and diabetes. Future research must be focused on synthetic ligands acting on SHP as a potential therapeutic target in a series of metabolic abnormalities. 

One important issue in lipidology is the understanding of the molecular mechanisms whereby cholesterol and fatty acids are absorbed from the intestine and are transported to the liver. The cholesterol absorption inhibitor ezetimibe can significantly reduce plasma total and LDL cholesterol concentrations by inhibiting the Niemann-Pick C1-like 1 protein (NPC1L1), an intestinal sterol influx transporter that can actively facilitate the uptake of cholesterol for intestinal absorption. The paper by O. de Bari et al. emphasizes the novel concept that, ezetimibe treatment also induces a complete resistance to two frequent metabolic abnormalities, namely, cholesterol gallstones and nonalcoholic fatty liver disease (NAFLD). Furthermore, it prevented hypercholesterolemia in mice on a Western diet. This model has high translational value and points to a key role for chylomicrons, the natural lipid carriers used by enterocytes to transport cholesterol and fatty acids into the body. The hypothesis that ezetimibe could prevent two prevalent hepatobiliary diseases (i.e., cholesterol cholelithiasis and liver steatosis) possibly through the regulation of chylomicron-derived cholesterol and fatty acid metabolism in the liver is discussed here.

Because several proteins are implicated in determining biliary lipid secretion in the liver and are regulated by several transcription factors, including nuclear receptors liver X receptor (LXR) and FXR, the paper by M. C. Vázquez et al. is focused on molecular mechanisms underlying the link between nuclear receptor function and the formation of cholesterol gallstones. A potent role for estrogen receptors in the pathogenesis of cholesterol gallstone disease, involving both genomic and nongenomic activation of signaling pathways, is discussed. Evidence in this respect is heavily supported by human and murine genetic, physiological, pathophysiological, and pharmacological studies. Indeed, expanding the knowledge about the role of NRs in gallstone formation will certainly lead to the discovery of novel and more effective therapeutic strategies in a typical example of a metabolic “mass disease,” that is, cholesterol cholelithiasis.

In the wide field of *lipopathy, *NAFLD is currently evolving as the most common liver disease worldwide, with potential costly and severe *sequelae, *including liver cirrhosis and hepatocellular carcinoma. In his paper, M. Fuchs underscored the concept that NAFLD not only represents an insulin resistance state characterized by a cluster of dysmetabolic cardiovascular risk factors, but also represents an independent risk factor for cardiovascular diseases. Of note, the bile acid-activated nuclear receptor FXR has been shown to play a role not only in bile acid but also in lipid (cholesterol and triglyceride) metabolism and glucose homeostasis. Specific targeting of FXR may be an elegant and very effective way to readjust dysregulated nuclear receptor-mediated metabolic pathways. Activation of FXR may result in not only beneficial actions but also potential undesirable side effects. One example is the (still unpredictable) balance between pro- and anti-atherogenic effects of FXR activation.

J. A. López-Velázquez et al. described the important role of several NRs in the liver as regulators of several critical metabolic steps involved in the pathogenesis of NAFLD. Such crucial steps include fat storage, export, uptake, oxidation, and lipolysis. A whole family of NRs is targeted by many ligands controlling lipid metabolism including fatty acids, oxysterols, and lipophilic molecules. Understanding the molecular mechanisms underlying the involvement of NRs in the pathogenesis of NAFLD may, therefore, offer targets for the development of new treatments of one of the most frequent chronic liver diseases worldwide. 

In their paper, R. Müllenbach et al. provided an update on genetic variants of NRs involved in regulating important aspects of liver metabolism. One such aspect is the application of NRs in genetic diagnosis of monogenic (Mendelian) liver diseases and their uses in clinical diagnosis. Moreover, a role of NR polymorphisms in common diseases can be anticipated, linking regulatory networks to complex and variable phenotypes. Technical advances contribute to the restless expansion of knowledge and include transgenic animal models, expression quantitative trait loci (eQTL) mapping, and genomewide association studies (GWASs). Thus, it is highly likely that personal genome information might eventually be able to predict a variety of risks associated with an individual's lifestyle such as high fat diet and alcohol as well as susceptibility to infectious liver diseases such as hepatitis B or C.

Menopause is a consequence of the normal aging process in women and it is thought that menopause is associated with a higher risk for cardiovascular diseases. Indeed, the post-menopause lipid profile is often altered, which represents a risk factor for cardiovascular diseases. The paper by P. J. Oliveira et al. reports on the mechanisms linking alterations of mitochondrial bioenergetics in the heart, as a consequence from normal aging and/or from the menopausal process, to decreased fatty acid oxidation and accumulation of fatty acid intermediates in the cardiomyocyte cytosol. Such lipotoxic consequences might represent the important link to increased cardiovascular risk in the menopausal women.

In conclusion, the field of lipidology has become even more complex and exciting when considering that the discovery of NRs and their pleiotropic functions have opened the way to multidimensional, multidisciplinary and translational studies. Since NRs are involved in virtually all physiological functions, understanding how NRs work is therefore essential to explain the complex pathophysiological mechanisms underlying liver and extrahepatic diseases. A new era in which NRs will represent valid therapeutic targets for several disorders is hopefully approaching. 

Lastly, we hope that this contribution will also help both young and experienced investigators in their daily difficult task to expand their research in the field of experimental and clinical lipidology in health and disease. 

*David Q.-H. Wang* *David Q.-H. Wang* *Brent A. Neuschwander-Tetri* *Brent A. Neuschwander-Tetri* *Piero Portincasa* *Piero Portincasa* *William M. Pandak* *William M. Pandak*

